# Genomes of the *Orestias* pupfish from the Andean Altiplano shed light on their evolutionary history and phylogenetic relationships within Cyprinodontiformes

**DOI:** 10.1186/s12864-024-10416-w

**Published:** 2024-06-18

**Authors:** Pamela Morales, Felipe Gajardo, Camilo Valdivieso, Moisés A. Valladares, Alex Di Genova, Ariel Orellana, Rodrigo A. Gutiérrez, Mauricio González, Martin Montecino, Alejandro Maass, Marco A. Méndez, Miguel L. Allende

**Affiliations:** 1 Millennium Institute Center for Genome Regulation, Santiago, Chile; 2 Departamento de Biología, Facultad de Ciencias, Universidad de Chile, Santiago, Chile; 3 Departamento de Ecología y Biodiversidad, Facultad de Ciencias de la Vida, Universidad Andres Bello, Santiago, Chile; 4 Laboratorio de Biología Evolutiva, Departamento de Ecología, Facultad de Ciencias Biológicas, Pontificia Universidad Católica de Chile, Santiago, Chile; 5 Grupo de Biodiversidad y Cambio Global (GBCG), Departamento de Ciencias Básicas, Universidad del Bío-Bío, Chillán, Chile; 6 DiGenoma-Lab, Instituto de Ciencias de la Ingeniería, Universidad de O’Higgins, Rancagua, Chile; 7 Centro de Modelamiento Matemático UMI-CNRS 2807, Universidad de Chile, Santiago, Chile; 8 ANID Millennium Institute for Integrative Biology (iBio), Santiago, Chile; 9 Facultad de Ciencias Biológicas, Pontificia Universidad Católica de Chile, Av Libertador Bernardo O’Higgins 340, Santiago, Chile; 10 Institute of Ecology and Biodiversity (IEB), Las Palmeras 3425, Ñuñoa, Santiago, Chile; 11 Bioinformatic and Gene Expression Laboratory, INTA-Universidad de Chile, Santiago, Chile; 12 Institute of Biomedical Sciences, Faculty of Medicine and Faculty of Life Sciences, Universidad Andres Bello, Santiago, 837001 Chile; 13 Laboratorio de Genética y Evolución, Departamento de Ciencias Ecológicas, Facultad de Ciencias, Universidad de Chile, Santiago, Chile; 14 Centro de Ecología Aplicada y Sustentabilidad (CAPES), Facultad de Ciencias Biológicas, Pontificia Universidad Católica de Chile, Santiago, Chile; 15 Cape Horn International Center (CHIC), Parque Etnobotánico Omora, Universidad de Magallanes, Puerto Williams, Chile; 16 Centro de Modelamiento Matemático IRL 2807 CNRS, Universidad de Chile, Santiago, Chile; 17 Departamento de Ingeniería Matemática, Universidad de Chile, Santiago, Chile; 18 Centro de Biotecnología Vegetal, Facultad de Ciencias de la Vida, Universidad Andrés Bello, Santiago, Chile

**Keywords:** Killifish, Pupfish, Cyprinodontiformes, Taxon sampling, Divergence times, South American Altiplano, Phylogenetics, Phylogenomics

## Abstract

**Background:**

To unravel the evolutionary history of a complex group, a comprehensive reconstruction of its phylogenetic relationships is crucial. This requires meticulous taxon sampling and careful consideration of multiple characters to ensure a complete and accurate reconstruction. The phylogenetic position of the *Orestias* genus has been estimated partly on unavailable or incomplete information. As a consequence, it was assigned to the family Cyprindontidae, relating this Andean fish to other geographically distant genera distributed in the Mediterranean, Middle East and North and Central America. In this study, using complete genome sequencing, we aim to clarify the phylogenetic position of *Orestias* within the Cyprinodontiformes order.

**Results:**

We sequenced the genome of three *Orestias* species from the Andean Altiplano. Our analysis revealed that the small genome size in this genus (~ 0.7 Gb) was caused by a contraction in transposable element (TE) content, particularly in DNA elements and short interspersed nuclear elements (SINEs). Using predicted gene sequences, we generated a phylogenetic tree of Cyprinodontiformes using 902 orthologs extracted from all 32 available genomes as well as three outgroup species. We complemented this analysis with a phylogenetic reconstruction and time calibration considering 12 molecular markers (eight nuclear and four mitochondrial genes) and a stratified taxon sampling to consider 198 species of nearly all families and genera of this order. Overall, our results show that phylogenetic closeness is directly related to geographical distance. Importantly, we found that *Orestias* is not part of the Cyprinodontidae family, and that it is more closely related to the South American fish fauna, being the Fluviphylacidae the closest sister group.

**Conclusions:**

The evolutionary history of the *Orestias* genus is linked to the South American ichthyofauna and it should no longer be considered a member of the Cyprinodontidae family. Instead, we submit that *Orestias* belongs to the Orestiidae family, as suggested by Freyhof et al. (2017), and that it is the sister group of the Fluviphylacidae family, distributed in the Amazonian and Orinoco basins. These two groups likely diverged during the Late Eocene concomitant with hydrogeological changes in the South American landscape.

**Supplementary Information:**

The online version contains supplementary material available at 10.1186/s12864-024-10416-w.

## Background

To understand how biodiversity has arisen is essential to disentangle the evolutionary history of species by reconstructing their phylogenetic relationships. The development of sequencing technologies has allowed the analysis of myriads of characters by obtaining the nucleotide sequences of genes. However, inconsistencies in the phylogenetic hypotheses proposed are often found, and these limitations can usually be explained by the selected genetic markers (usually mitochondrial genes). Furthermore, the use of only a few genes is not enough to provide statistical support for every node in a phylogeny [1–3]. Genome-wide analyses alleviate this problem by considering hundreds to thousands of genes [1]. Therefore, the accumulation of genetic and genomic information in databases has opened up the opportunity to evaluate and/or re-evaluate the relationship of several taxa that were previously estimated based on limited or incomplete information [4].

The Cyprinodontiformes, also known as killifish, is a large and diverse order of fresh- and brackish water species with a temperate and tropical distribution [5]. The phylogenetic relationships in this order have been subject to multiple systematic changes. Although there are studies on representatives of the order, the origin and early diversification of the South American species have not received sufficient attention. Parenti (1981) [5] performed the first cladistic analysis for this group, based on osteological characters, defining two suborders: Aplocheiloidei and Cyprinodontoidei. At present, these suborders comprise three and eleven families, respectively [6]. Several studies have attempted to infer the phylogenetic relationships between and within these families [7–11]. Particularly, the family Cyprinodontidae sensu Parenti (1981) [5] included genera from North, Central and South America, as well as members from the Mediterranean and Anatolian area. Parenti (1981) [5] proposed a close relationship between the genera *Orestias* and *Aphanius*, which was ratified later by Parker & Kornfield (1995) [12]. The latter estimated the phylogeny considering 16 members of this family and using partial mitochondrial sequences (16S and the control region). However, this proximate phylogenetic position between both groups contrasts with their highly distant geographical location: *Aphanius* is distributed in the Mediterranean, Red Sea and the Persian Gulf basins (Paleartic region), while *Orestias* is distributed across the South American Altiplano (Neotropical region). Subsequent studies based on molecular phylogenies have challenged some of the relationships established by Parenti (1981) [5]. These studies have suggested that Cyprinodontidae is a polyphyletic group because *Aphanius*, *Cubanichthys* and *Orestias* genera would not share a common ancestor, and they would not be related to the species from North and Central America [8–10]. Later, *Aphanius* was established as a different family, Aphaniidae, also based on morphological data [13]. Altogether, this body of evidence indicates that the phylogenetic position of the *Orestias* is, at the very least, unresolved, while strongly suggesting that it may be positioned outside the Cyprinodontidae family.

The genus *Orestias* comprises 46 species inhabiting lakes, rivers, tributary streams, lagoons and springs, between 2,000 and 4,500 m above sea level (m a.s.l.), across the Altiplano covering territories of Bolivia, Peru and Chile; half of the species are found in Lake Titicaca [14]. The Altiplano basin is characterized by an arid to semi-arid climate, with a short rainy season known as the South American summer monsoon (SASM; [15]. However, climatic conditions have shown historical variability, given the existence of successive paleolakes that occurred during the Late Pleistocene that connected currently isolated hydrological systems [16–19]. These extensive paleolakes could explain, at least partly, the *Orestias* distribution in the Altiplano.

In this study, we confronted the problem of the phylogenetic position of the genus *Orestias* within the order Cyprinodontiformes using a multi-faceted approach. To tackle phylogeny reconstruction, we applied two approximations: we considered (i) a small set of taxa with many genes and (ii) an extensive taxa representation but a few genes [20]. For the first approach, we constructed a phylogenomic tree with all the species of the order that have available genomes to date. We used whole genome sequencing to add three new *Orestias* genomes (*O. gloriae*, *O. laucaensis* and *O. chungarensis*) and included the recently reported genome of *O. ascotanensis* [21]. After assembly and annotation of the genomes, we detected a number of interesting features in the *Orestias* genomes, such as a reduced size, a consequence of a lower content of transposable elements (TEs) and common patterns of TE activity, evidenced by similar distributions of Kimura distances. These features are notably different from what is observed in other closely related families, such as Cyprinidontidae and Anablepidae. TEs have been previously associated with relevant evolutionary events such as speciation [22], karyotype variability [23], evolutionary innovations [24], and other processes [25]. For the second approach, we reconstructed the phylogeny of the order by performing a stratified taxa sampling [20] comprising at least one species of almost every genus of every family of the order. For this approach, we used available nucleotide sequences for 12 genes (four mitochondrial and eight nuclear genes). We also extracted the gene sequences from the available genomes, to compare the phylogenomic and phylogenetic results of both approaches.

Overall, our results clearly reveal that the genus *Orestias* is not part of the family Cyprinodontidae sensu Parenti (1981), but represents the rediscovered family Orestiidae Bleeker (1859) [26]. The Orestiidae are constituted by the *Orestias* and possibly by the *Pseudorestias* genera. We infer that *Orestias* would have diverged from their South American sister group during the Eocene–Oligocene transition, driven by hydrogeological changes that occurred in South America [27], becoming isolated in the Altiplano where it further diversified.

## Results

### Genome assembly and annotation of three *Orestias* species

We sequenced and assembled the genome of three *Orestias* species from three Chilean localities (Southern Altiplano) [Supplementary 1: Table S1]. Briefly, our strategy was, first, to generate de novo assemblies for each species, and then to perform reference-guided scaffolding based on the recently published genome of *O. ascotanensis* [21], which remarkably improved the quality metrics of the new genome assemblies. For instance, the three new genomes showed a nearly 42-fold improvement in the length of the N50 parameter, and the number of complete orthologs estimated by BUSCO (Benchmarking Universal Single-Copy Orthologs) rose nearly 12% in all the species [Supplementary 1: Table S2]. Hence, the final assembly lengths for these three new genomes are approximately 700 Mb (*O. gloriae* is 694.8 Mb, *O. laucaensis* is 700.6 Mb and *O. chungarensis* is 698,3 Mb), which is similar to the reference genome of *O. ascotanensis* (696.3 Mb; 21). Finally, gene annotation shows that the four sequenced *Orestias* genomes have a very similar number of predicted genes (*O. ascotanensis*, 33,429; *O. gloriae*, 31,270; *O. laucaensis*, 31,026; *O. chungarensis*, 31,168) [Supplementary 1: Table S1].

### Comparison of the repetitive content in *Orestias* genomes

We also explored the assembled genomes in terms of their repetitive content and the diversity of transposable elements (TEs) they harbor. We decided to incorporate the genomes of *Anableps anableps* (genome size = 867 Mb) and *Cyprinodon variegatus* (genome size = 1.03 Gb) into these analyses because they are close relatives of *Orestias* with available sequenced genomes (see below). Overall, the repetitive content is quite similar in all *Orestias* species [Supplementary 1: Figure S1]. For instance, 22.27% of the genome of *O. ascotanensis* is composed of repetitive sequences, while in *O. chungarensis* it is 24.21%, in *O. laucaensis* it is 24.33%, and in *O. gloriae* it is 24.18%. However, the repetitive content of the *Orestias* species is proportionally smaller than the other two species evaluated, *A. anableps* (40.12%) and *C. variegatus* (30.78%). It is worth noting that the lower repetitive content in *Orestias* species coincides with smaller genome sizes and a lower overall content of TEs.

In terms of the co-occurrence of TEs across the species, we found that 38 superfamilies are shared among the four *Orestias* species analyzed [Supplementary 1: Figure S2A], and 34 of them are also shared with *A. anableps* and *C. variegatus* [Supplementary 1: Figure S2B], with three superfamilies being exclusive for the *Orestias* species (Dada, ERVL-MaLR, and MuLE-NOF).

In order to quantify the different contributions of the shared superfamilies from each order of TEs and non-repetitive regions to the genome size, we performed a pairwise comparison using Log_2_ fold-change (Log_2_FC), to compare every species against each other [Supplementary 1: Figure S3].

Our results show that most of the variation in genome size could be explained by changes in the content of repetitive elements, contrary to non-repetitive regions. Moreover, DNA elements and short interspersed nuclear elements (SINEs) appear as the orders with the most significant differences in terms of the number of bases. Finally, in terms of relative contribution to the genome size, *Orestias* species show a higher proportion of the interspersed nuclear elements (LINE) and long terminal repeats (LTR) orders, likely due to their lower absolute content of DNA elements in these genomes [Supplementary 1: Figure S4].

In addition, we explored the distributions of Kimura distance values for all these species in order to explore the temporal dimension of the activity of TEs [Supplementary 1: Figure S5]. This revealed shared evolution and amplification events in the main orders of TEs in the *Orestias* species (overlapping curves), which is drastically different from what is observed in the Kimura distributions of *C. variegatus* and *A. anableps*. This, together with the Log_2_FC analysis, suggests that SINE and DNA elements might be responsible, or at least relevant factors, for the reduced genome size observed in the *Orestias* species.

### Phylogenomic analysis of the Cyprinodontiformes order

**Fig. 1:**
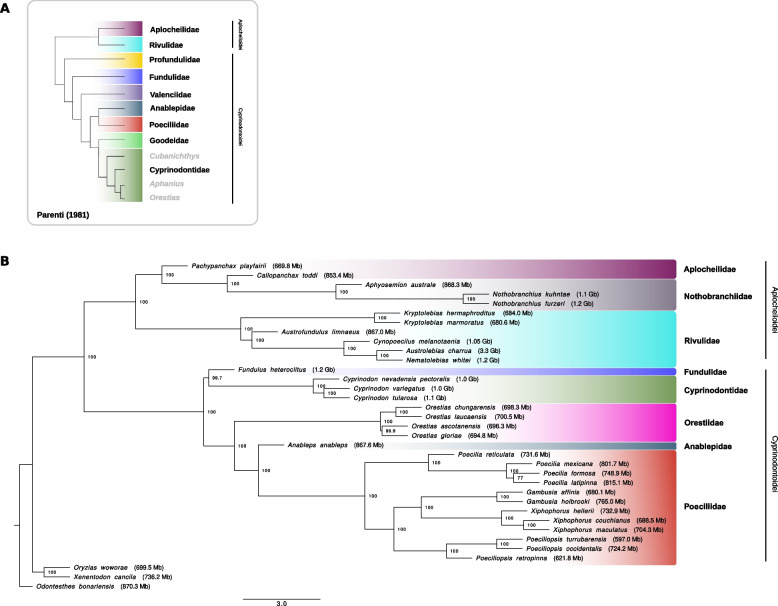
Phylogenetic trees of the Cyprinodontiformes order based on morphological characters and genomic information. **A** Phylogenetic relationship of the families within the Cyprinodontiformes order based on morphological characters. The Cyprinodontidae family includes the genera *Orestias*, *Cubanichthys* and *Aphanius*. Family names are indicated in coloured boxes, and the sub-orders are indicated with a black line at the right. Modified from Parenti (1981). **B** ASTRAL species tree for the Cyprinodontiformes order. Reconstruction is based on 902 single-copy orthologs shared by the 32 available genomes for the order, including the three *Orestias* genomes reported in this study, and the three outgroups. The genus *Orestias* is indicated as a member of the Orestiidae family (see details in the text). Quartet bootstrap support is indicated for each internal branch. The genome size of each species is indicated in parenthesis after the species name. Family names are indicated in coloured boxes on the right and the sub-orders are indicated with a black line at the right

Using BUSCO we detected 902 complete single-copy ortholog genes that are shared by the 35 available fully sequenced genomes within the order [Supplementary Table S6]. This dataset was used for the species tree reconstruction to estimate the phylogenetic relationships at the genomic scale (Fig. 1). We recovered every lineage within the order as a well-supported group with quartet bootstrap supports ranging from 99.7 to 100, (except for the group formed by *Poecilia formosa* and *P. latipinna* that has a support of 77). Within the Aplocheiloidei suborder, the families Aplocheilidae and Nothobranchiidae are sister groups, closely related to the family Rivulidae. Notably, and in concordance with previous studies mentioned above, this phylogenomic approach did not recover the Cyprinodontidae family sensu Parenti (1981) [5] as a monophyletic group. Although *Orestias* is not closely related to the *Cyprinodon* genus, our phylogenomic analysis revealed a strong and well-supported relationship with the Poeciliidae and Anablepidae families. In contrast, the *Cyprinodon* genus is more closely related to the *Fundulus* genus. These results suggest that there is a close correlation between evolutionary and geographical distance within the Cyprinodontiformes. In this sense, *Orestias*, which is found in the South American Altiplano, is closely related to other South American killifish families. On the other hand, *Cyprinodon* is a sister group to the Fundulidae family, both distributed in North and Central America.

### Phylogenetic relationships in the Cyprinodontiformes order

**Fig. 2:**
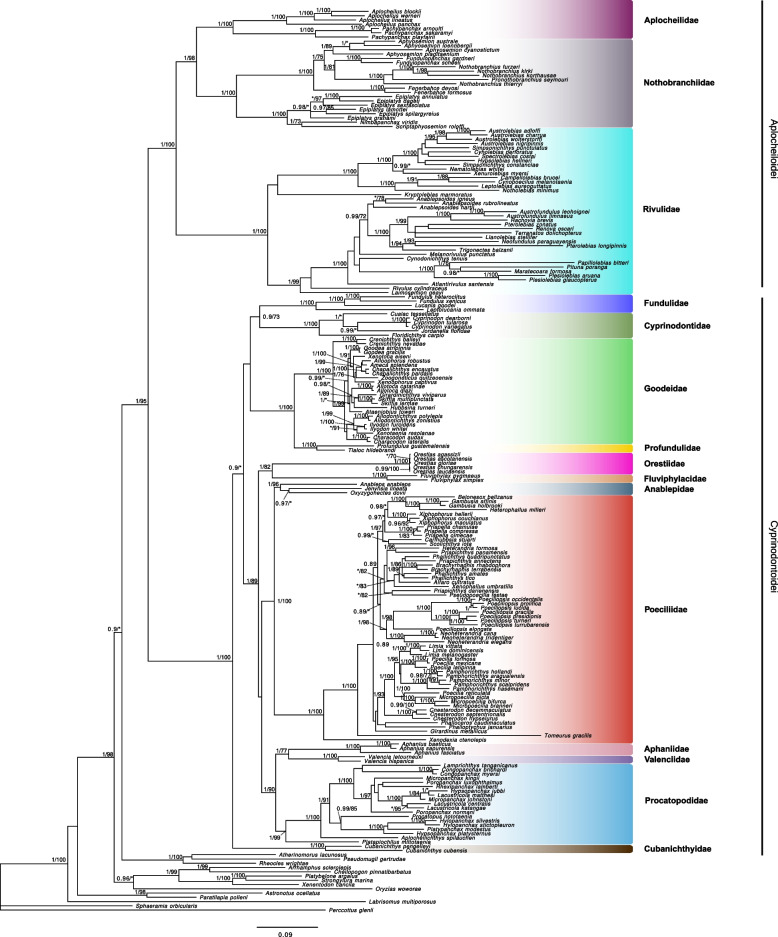
Maximum-likelihood and Bayesian reconstruction tree for the Cyprinodontiformes order. Phylogenetic analyses based on 12 molecular markers (four mitochondrial genes, *16S*, *cox1*, *nd2* and *cytb*, and eight nuclear genes, *rag1*, *glyt*, *sreb2*, *rho*, *enc1*, *sh3px3*, *myh6* and *x-src*) for representatives of almost every genus within each family of the Cyprinodontiformes order. The genera *Orestias* and *Cubanichthys* are indicated as members of the Orestiidae and Cubanichthyidae families, respectively (see details in the text). Node labels indicate bootstrap support (> 70) and posterior probability (> 0.95). Family names are indicated in colored boxes and the sub-orders are indicated with a black line at the right

We applied stratified taxa sampling to construct a more comprehensive phylogeny that covered a wider range of taxa. This involved selecting one species from most genera within each family of the order, as well as many available nucleotide sequences, in order to construct a more taxonomically representative phylogeny [Supplementary Table S7]. Using this dataset, we constructed a phylogenetic tree using 12 concatenated nucleotide sequences, four mitochondrial genes and eight nuclear genes, for 198 species belonging to the Cyprinodontiformes (Fig. 2). We extracted the four mitochondrial markers (*16S*, *cox1*, *nd2* and *cytb*) for the *Orestias* species from their de novo assembled mitogenomes. We obtained a mitochondrial genome of 16,650 bp for *O. gloriae* with a GC content of 44.1%, and 16,603 bp and a GC content of 44.2% for both *O. chungarensis* and *O. laucaensis*. All assembled mitogenomes sequences contain both the small (*16S*) and large (*23S*) ribosomal subunits, 13 coding genes (*nad1*, *nad2*, *cox1*, *cox2*, *atp8*, *cox3*, *nad3*, *nad4L*, *nad4*, *nad5*, *nad6* and *cytb*), 23 tRNAs (being Phenylalanine, Leucine and Serine the redundant tRNAs) and the Control region (*D-Loop*) [Supplementary 1: Figure S6]. Additionally, when we compared the four *Orestias* species, we found that these mitogenomes are highly similar, with an average identity of 99.15% between them.

Both Maximum Likelihood (ML) and Bayesian Inference (BI) methods, retrieved congruent phylogenies that showed identical topologies. In accordance with the phylogenomic tree (Fig. 1), we recovered the order, the two suborders, and every family as well-supported groups, except for the family Cyprinodontidae sensu Parenti (1981) [5], which was recovered as a polyphyletic group. The main lineages were: (i) *Orestias* showing a close relationship with the genus *Fluviphylax* Whitley, 1965, another South American group distributed in the Amazonian and Orinoco basins, which constitute the monotypic family Fluviphylacidae Roberts 1970 [11]. (ii) *Cyprinodon*, *Cualac*, *Jordanella* and *Floridichthys* formed a clade that is closely related to the family Fundulidae (similarly to the phylogenomic tree). In turn, these are closely related to a group formed by the families Goodeidae and Profundulidae. (iii) *Aphanius* appears as the sister group of the family Valenciidae, both Old World groups. This clade shows to be closely related to the family Procatopodidae, the African lampeyes, and these three (*i.e.*, ((*Aphanius*, *Valencia*), *Procatopodidae*)) are the sister group of the lineage formed by the Anablepidae and Poeciliidae families. Furthermore, this clade, containing New and Old World families, is closely related to the group formed by *Orestias* and *Fluviphylax*. Finally, (iv) the genus *Cubanichthys*, distributed in the Caribbean islands (Cuba and Jamaica), was recovered in a different lineage within the suborder Cyprinodontoidei.

For the Aplocheiloidei suborder, we were also able to recover its three families with the same relationship we found in the phylogenomic approach: the Aplocheilidae and Nothobranchiidae are sister families and are closely related to the Rivulidae family.

### Divergence time estimation

**Fig. 3:**
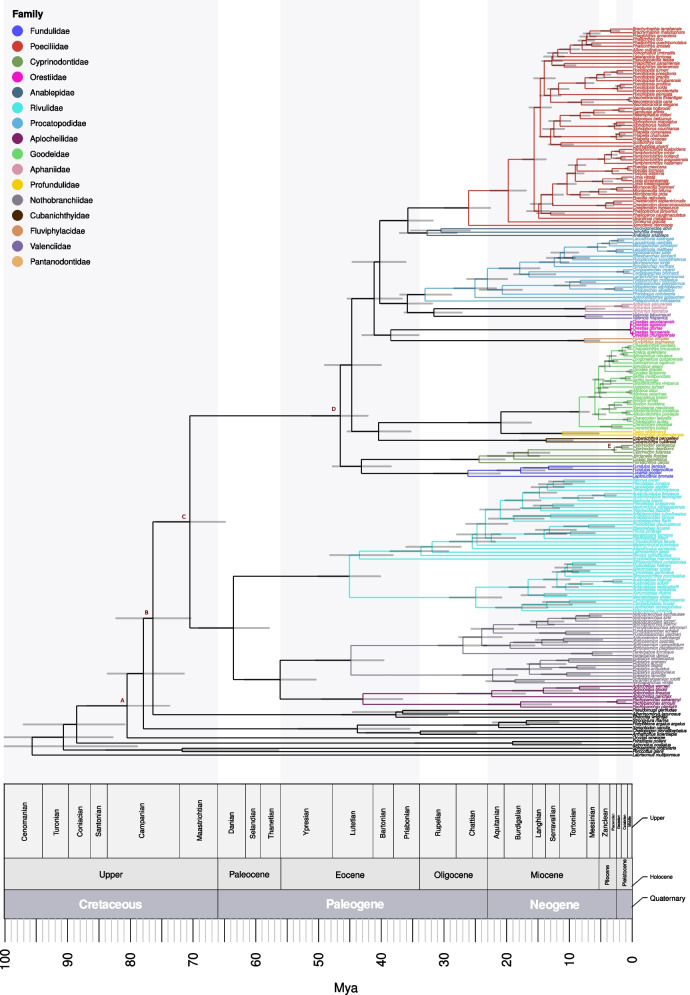
Calibrated phylogeny of the Cyprinodontiformes order. Bayesian inference of the divergence time of each node. Node bars show the 95% HPD of the divergence times. The color of the branches indicates the different families. Eras, periods and epochs are indicated for the last 100 Mya. Letters A to E marks the nodes used for the calibration of the phylogeny. Details of these calibrations are in Supplementary 1: Table S3

The calibrated phylogeny generated is shown in Fig. 3. We estimated the age of the divergence time of the Cyprinodontiformes order at 70.41 (64.81–75.79) million years ago (Mya), in the Late Cretaceous (Maastrichtian age). On the other hand, the suborder Aplocheiloidei may have diversified in the Early Paleocene about 63.48 (57.84–68.94) Mya, while the suborder Cyprinodontoidei yielded an age estimate at 46.47 (42.12–51.06) Mya, in the Middle Eocene. Interestingly, most of the divergence events that gave rise to the Cyprinodontoidei families occurred during the Middle Eocene to Early Oligocene. The *Orestias* genus showed an estimated divergence time from the genus *Fluviphylax* at about 38.44 (34.03–42.97) Mya, during the Late Eocene, while the diversification of each genus occurred much later: 312.15 (168.02–455.46) Kya for *Orestias* and 7.67 (5.23–10.24) Mya for *Fluviphylax*. On the other hand, the Cyprinodontidae family (genera *Cyprinodon*, *Jordanella*, *Cualac* and *Floridichthys* in this study) diverged from its sister family Fundulidae at 43.09 (38.43–47.68) Mya, shortly after the estimated age of the suborder. Furthermore, the genera *Aphanius* and *Valencia* showed a diversification age estimate in the early Oligocene, about 31.58 (27.15–36.23) Mya.

## Discussion

### New available *Orestias* genomes

In this study, we sequenced and assembled three new genomes of the *Orestias* genus, at the scaffold level using only short read technology. The previously sequenced *O. ascotanensis* genome proved to be a useful reference for de novo assembly [28] due to the close phylogenetic relationships among congeneric species, facilitating the genome assemblies of *O. gloriae*, *O. laucaensis* and *O. chungarensis* [Supplementary 1: Table S1]. As a measure of the improvement provided by the reference genome, the degree of completeness of the new species’ scaffolds increased, raising the percentage of complete genes from 77.3% to 89%, on average. This allowed us to be able to select from a larger number of suitable genes as molecular markers for the phylogenomic approach.

Hence, at present, the available genomes of the *Orestias* genus comprise two ecologically contrasting species pairs: the first is *O. ascotanensis* and *O. gloriae*, which inhabit similar brackish waters of two high-altitude salt pans, Ascotan [29] and Carcote [30], respectively. The second corresponds to the freshwater-living species, *O. laucaensis* and *O. chunganrensis*, present in a river (Lauca River; [31]) and a lake (Lake Chungara; [32]), respectively. The divergence time between the species of each pair is different: the freshwater species diverged 12–8 Kya [33, 34], while the salt pan species have likely diverged 280 Kya, according to our results. Thus, genomic comparisons between these species represent a suitable model to study the genomics of speciation [35–37] and the effect of different selection pressures given their highly contrasting environments.

### The reduction in the genome size of the *Orestias* species

An interesting observation that arose from the genome assemblies generated for the *Orestias* species is that their genome sizes (mean = 697.4 Mb) are significantly smaller than the average of the 35 species analyzed in our phylogenomic study (mean = 895.6 Mb; sd = 453.17). Moreover, within the Cyprinodontoidei suborder, *Orestias* is only surpassed by *Poeciliopsis* and *Xiphophorus* as the genera with the smallest genomes (Fig. 1). In this regard, our analysis using Log_2_FC showed that the difference between the genome sizes of the *Orestias* species and their close relatives (*C. variegatus* and *A. anableps*) can be mainly explained by changes in the content of DNA elements and SINEs. One of the few comparative studies addressing the matter of genome size variation and TE content in fishes [38] also found differences in genome size mainly due to differences in the content of repetitive DNA elements in zebrafish, medaka, stickleback, and tetraodon. However, considering the rather tenuous relationship between these species (they all belong to different orders), it is difficult to establish the direction of the genome size change and thus to assess whether the observed differences are a consequence of contractions or expansions of DNA transposons. Here, we considered a group of much more closely related species, with *Orestias* as the genus displaying one of the smallest genome sizes. Therefore, a reduction in genome size, driven by a decrease in the content of DNA transposons and SINEs, is the most parsimonious explanation, rather than an increase in genome size in all the other species. Di Genova et al. (2022) [21] also reported on several gene families in the *O. ascotanensis* genome that have suffered recent contraction. In this sense, our results support the hypothesis of an overall reduction in DNA content affecting genes and TEs. It is scientifically compelling to further explore this issue by progressively enlarging the available genomic information on the Cyprinodontiformes. This will allow not only to improve our understanding of the dynamics of the genome size changes but also to define how the different orders of TEs have changed during the evolution of this group.

### Phylogenetic position of the genus *Orestias* and the taxonomic implications for the family Cyprinodontidae and the order Cyprinodontiformes

By using both phylogenomic and phylogenetic approaches, we accurately determined the position of the *Orestias* genus within the Cyprinodontiformes order and revealed that the Cyprinodontidae family sensu Parenti (1981) [5] is not a natural group, a finding which is consistent with previous molecular phylogenies [8–10]. These results demonstrate how genomic information can improve the resolution of phylogenetic reconstructions by providing strong statistical support for an entire phylogenomic tree (Fig. 1; 1–3). In addition, our stratified sampling regime [20], which included representatives of almost every genus and family in the order, enabled us to uncover previously undescribed phylogenetic relationships. (Fig. 2). Specifically, we found that the genus *Orestias* is not related to the Cyprinodontidae family sensu Parenti (1981) [5], but rather to the South American genus *Fluviphylax*, the only genus belonging to the family Fluviphylacidae. These two genera are closely related to the group comprising the Poeciliidae and Anablepidae families, as previously reported by Bragança & Costa, (2018, 2019) [11, 39] who only considered *Fluviphylax*, but also to Old World families such as Procatopodidae, Aphaniidae and Valenciidae. Notably, our study discovered relationships among New and Old World families that are consistent with their geographical proximity. We found that *Orestias* is not related to the Old World genus *Aphanius*, as proposed by Parenti (1981) [5], but rather to another South American group, the family Fluviphylacidae. In contrast, *Aphanius* is closer to *Valencia*, both distributed in the Western Palaearctic [26], and phylogenetically more proximate to the family Procatopodidae, which includes the African lampeyes. Overall, our study sheds new light on the evolutionary relationships within the Cyprinodontiformes order and provides important insights into the biogeography and natural history of these families.

Importantly, our findings support the proposal made by Freyhof et al., (2017) [26] whom consider the *Orestias* genus as belonging to the forgotten family Orestiidae Bleeker, 1859 and, thus, should not be included as a member of the Cyprinodontidae any longer. Similarly, the *Cubanichthys* genus should now be integrated into the family Cubanichthyidae Parenti, 1981. Furthermore, the composition of the Cyprinodontidae family should now be restricted to its North and Central American members (*i.e.*, the genera *Cyprinodon*, *Floridichthys*, *Cualac, Jordanella*, and possibly *Megupsilon* and *Garmanella)* [Supplementary 1: Figure S7]. As a result, the suborder Cyprinodontoidei would harbour thirteen families, with almost a third of them being monotypic (i.e., Valencidae, Fluviphylacidae, Cubanichthyidae and Pantandontidae).

Our study includes the most extensive taxon sampling for the Cyprinodontidae family to date. However, it is still incomplete because we did not include the three remaining genera due to a lack of genetic information. Specifically, there are no nuclear sequences for *Megupsilon* Miller & Walters 1972, *Garmanella* Hubbs, 1936 and *Pseudorestias* Arratia, Vila, Lam, Guerrero & Quezada, 2017. Despite this limitation, we hypothesize that their phylogenetic position is likely based on their geographical distribution: *Megupsilon aporus*, the only species of its genus, was found in Mexico (Nuevo León) and is now listed as extinct according to the IUCN Red List [40]. *Garmanella* is distributed in Mexico and Belize, and are therefore probably related to the North and Central American cyprinodontids. *Pseudorestias*, which is found in the Altiplano area, proximate to the habitat of *Orestias* species, is probably a sister genus [41]. Nevertheless, further studies are necessary to confirm our hypothesis. Additionally, we did not include the Pantandontidae family in this study due to the ongoing debate about its phylogenetic position [8, 11]; however, the inclusion of genomic information of this species would be a valuable resource to resolve the issue.

In 1981, Parenti [5] established the family Cyprinodontidae based on an extensive anatomical and osteological analysis of the Cyprinodontiformes order. Parenti used three diagnostic traits to define the family: i) “the dorsal processes of the maxillaries expanded medially nearly meeting in the midline, and possessing a distinct groove; ii) the lateral arm of the maxilla greatly expanded; and, iii) the tooth plate of the fourth pharyngobranchial greatly reduced”. Additionally, most of the species in this group inhabit shallow, brackish waters, and many exhibit a high degree of salinity tolerance. The inconsistency between the morphology-based classification and the molecular phylogenies suggests that the family Cyprinodontidae sensu Parenti (1981) [5] may be a good example of convergent evolution, where morphological similarities could be adaptations to these extreme environments [42–44]. Nonetheless, further research is required to comprehend how these environmental characteristics might have influenced the similarities in osteological traits.

Broadly, our results concur with previous phylogenetic studies, supporting the monophyly of the Cyprinodontiformes order and its two suborders, Aplocheiloidei and Cyprinodontoidei [5, 7, 8, 10]. We also recovered the three well-known families within the suborder Aplocheiloidei and confirmed their relationships [8–10, 45]: Aplocheilidae and Notobranchiidae are sister groups, closely related to the family Rivulidae (Figs. 1 and 2). Each family has a specific geographic distribution [Supplementary 1: FigureS7]: Aplocheilidae is found along the Indian Ocean, Nothobrachiidae in Africa and Rivulidae in South America. For the suborder Cyprinodontoidei, in addition to Cyprinodontidae, we obtained strong support for the remaining families, which are Fundulidae, Profundulidae, Goodeidae, Anablepidae, Fluviphylacidae, Poeciliidae, Aphaniidae, Valencidae and Procatopodidae. However, some relationships between them are updated, as mentioned above [8–10].

### Divergence time of the order Cyprinodontiformes, the suborders Aplocheiloidei and Cyprinodontoidei, and the family Orestiidae

Our results for the divergence ages for the Cyprinodontiformes order (70.4 Mya) and its suborders Aplocheiloidei (63.5 Mya) and Cyprinodontoidei (46.5 Mya) agree with previous molecular [46, 47] and fossil-based [48] calibrations. Our estimations indicate that the diversification of Cyprinodontiformes occurred during the Maastrichtian age when global sea levels increased, leading to several marine transgressions that flooded the coastal environments [49–53]. Capobianco & Friedman (2019) [48] also hypothesized that long-distance dispersal allowed the colonization of distant new environments, which could explain the distribution of cyprinodontids across several continents [Supplementary 1: Figure S7].

The divergence of the two sub-orders, Aplocheiloidei and Cyprinodontoidei, occurred after the Cretaceous-Paleogene boundary, a period marked by a significant shift in global biodiversity due to the Chicxulub impact [54, 55]. While this caused one of the five largest mass extinctions, leading to the disappearance of ~ 70% of species including the non-avian dinosaurs [54, 55], several taxa were less affected and were able to diversify and thrive, including mammals, birds, worm lizards, spiny-rayed fishes and freshwater species [56–61]. In particular, freshwater environments acted as a refuge for biota due to their higher thermal inertia, and because the detritus-based food web of riverine systems was less affected than terrestrial and marine primary production [56, 60, 62–64]. This would allow them to occupy ecological roles left by extinct forms and thus diversify [57, 58]. Later, the suborder Cyprinodontoidei diversified (46.5 Mya) during the early-middle Eocene (specifically after the Ypresian/Lutetian boundary). Marine transgressions and regressions also characterized this period, which could have helped the long-distance dispersal of their ancestor, similarly to how the order’s ancestor did [48, 49, 65].

We estimated the divergence time of the clade comprising *Orestias* and *Fluviphylax* to be 38.4 Mya, which coincides with significant hydrogeological changes in South America that impacted the diversification of its ichthyofauna [27]. The Eocene divergence of *Orestias* and *Fluviphylax* aligns with the end of the initial uplift of the Central Andes at 65 to 34 Mya [66–68]. We hypothesize that, before this event, their ancestor was distributed in north-western South America. After the Andean uplift, the ancestral distribution could have been separated into two areas, a high-elevation Andean distribution and a low-elevation distribution in the Proto-Amazon-Orinoco system. The ancestor colonizing the Andes then could have dispersed southward to the rest of the Altiplano, eventually diversifying into several *Orestias* species. Our estimation for the diversification time of *Orestias* is 312.2 kya, but this estimation must be taken with caution because it considers only five out of 46 described species, four of which are endemic to the Chilean Altiplano. Therefore, we clearly have not covered the wide diversity of habitats and geographical range of this genus. Cassemiro et al. (2023) [27] determined that the diversification of *Orestias* represents one of the few rapid shifts in the diversification rate of South American fish fauna, as it would have happened ~ 7 Mya. However, this time estimation must also be taken cautiously as this report only considered mitochondrial markers for the 19 *Orestias* species analyzed. This limitation led to an unsupported *Orestias* lineage (bootstrap support is 61) and did not clarify the relationship with the sister groups.

In the case of the ancestor that remained in the Proto-Amazon system, it could have inhabited the Pebas system, a large wetland and swamp system in the Western Amazon, from 23 to 8 Mya [69, 70]. Subsequently, a rapid uplift of the Northern Andes occurred from 11 to 9 Mya, modifying the drainage network and changing the flow direction of the Amazonian River from northward to eastward and transforming it into a fluvial system at 7 Mya [69–72]. This coincides with our estimated divergence time for *Fluviphylax* (7.67 Mya), indicating that its ancestor would have diversified in the Orinoco basin during this period. Our estimation agrees with that obtained by Bragança & Costa (2018) [11], who estimated a divergence time between *F. simplex* and *F. pygmaeus* (the same species we considered here) at 6.5 Mya, probably related to the palaeogeographical events that formed of the Amazonian basin.

We would like to highlight that the divergence between *Orestias* and *Fluviphylax* occurred approximately 38 million years ago, whereas the diversification of each genus occurred much later, at around 7 million years ago. One possible explanation for the absence of intermediate forms between these two clades is that they went extinct, possibly when the Pebas system disappeared. Alternatively, these intermediate forms could still exist, but they are not yet sampled and/or there is no genetic information from them. However, this seems unlikely given the extensive knowledge of South American freshwater fish fauna obtained to date [27, 73–75].

## Conclusions

This study aimed to determine the phylogenetic position of *Orestias* within the Cyprinodontiformes order using two different approaches: a phylogenomic analysis with a small set of taxa and many genes and a phylogenetic analysis with a large taxa set and few genes. The study found that *Orestias* has an evolutionary history linked to the South American fish fauna, contrary to what was previously inferred from morphological characters. The study also supports the suggestion that *Orestias* (and possibly *Pseudorestias*) form the family Orestiidae (an abandoned family name that we recover), and that it should no longer be considered a member of the Cyprinodontidae family. Additionally, we found that the Orestiidae and Fluviphylacidae families are sister groups, which was possible to detect by including representatives of every family of the order in a stratified taxa sampling. This is the first report of this relationship, which has gone unnoticed in previous phylogenetic analyses.

On the other hand, the newly sequenced genomes from three different *Orestias* species were highly useful in improving the understanding of the evolution of this genus. Firstly, the statistical support of our phylogenomic analysis increased significantly compared to the phylogenetic result due to the higher number of characters used. Secondly, we found that the smaller genome sizes of *Orestias*, compared to the other species of the order, could have been the result of a contraction in TEs. This is a noteworthy contribution to the study of the evolution of genomic features in a phylogenetic framework. Therefore, our study highlights the importance of genome sequencing of species from genera or families with no such available information. This allows the re-evaluation of the phylogenetic relationships of highly distant species, increases the certainty of phylogenetic inference, and helps to reveal the evolution of a group and their genomes.

Finally, our study emphasizes the importance of updating and curating both genetic and taxonomic databases to take advantage of the huge amount of accumulated information in modern-day biological research.

## Methods

### Sampling and sequencing

Briefly, one adult female from each of the three *Orestias* species was sampled between 2013 and 2016. They were collected from previously described Chilean populations: *O. gloriae* was collected in the Spring 1 of the Carcote salt pan (21°17′02.1’’S; 68°19′29.7’’W); *O. chungarensis* was sampled in Lake Chungará (18°15′58.4’’S; 69°09′38.3’’W) and *O. laucaensis* in the Lauca River (18°11′36.4’’S; 69°16′23.4’’W). All individuals were euthanized via immersion in an overdose of tricaine methanesulfonate (MS-222) and were left there for 10 min after the cessation of opercular movement [76]. Samples were preserved independently in RNAlater solution (Thermo Fisher Scientific) and kept at -80 °C until DNA isolation for sequencing. Genomic DNA was isolated using the Dneasy Blood & Tissue kit (Qiagen), according to the manufacturer’s instructions and performing a Rnase A (Qiagen) treatment. Later, a shotgun library (2 × 150 paired-end reads) was constructed and genome sequencing was carried out in the Illumina HiSeq 2500 platform by Macrogen Inc. (Seoul, Republic of Korea) (See the *“Availability of data and materials”* section).

### Genome assemblies and annotation

For the assembly of the genomes of *O. gloriae*, *O. chungarensis* and *O. laucaensis* we followed a combined strategy. First, we generated contig-level genome assemblies for each species using DISCOVAR de novo assembler (2017 release) [77]. Then, we performed a reference-guided scaffolding using Ragoo v1.1 [78], and the recently published genome of *Orestias ascotanensis* as a reference [21]. The resulting assemblies were then evaluated using BUSCO v4.0.6 [79] and annotated by mapping the *O. ascotanensis* annotation using LiftOff v1.5.1 [80].

The mitochondrial genomes of *Orestias gloriae*, *O. chungarensis* and *O. laucaensis* were assembled from the genomic paired-end reads, using the Norgal pipeline v1.0.0 [81]. This methodology allows us to extract mitochondrial reads from genomic sequencing and to perform a de novo assembly for this organelle’s genome. The mitogenome of *O. ascotanensis* [82] was also used to identify mitochondrial genes. All mitogenomes were annotated using MitoAnnotator, from the MitoFish database [83].

### Annotation and classification of repetitive elements

The annotation of the repetitive elements was performed in the four *Orestias* species included in this work, together with two additional species: *Anableps anableps* (accession number: GCA_014839685.1) and *Cyprinodon variegatus* (accession number: GCF_000732505.1), due to their having available genomes and that they are closely related to the *Orestias* genus, according to our phylogenetic analysis.

First, we used RepeatModeler [84] to identify novel families of repetitive elements in each species. Then, we classified these families using a complementary approach involving RepeatClassifier (the default classification method of RepeatModeler; [84], and TERL [85], a deep-learning-based method. Our strategy was to train and test a classifier based on the *non-unknown* predictions of RepeatClassifier, and then we used this model to further extend the annotation of unknown elements. With the goal of generating representative sets for Cyprinodontiformes, we considered a panel with the aforementioned species, together with predictions performed on several species from the Aplocheiloidei suborder (for instance, *Austrolebias charrua*, *Cynopoecilus melanotaenia*, *Austrofundulus limnaeus*, *Nothobranchius furzeri*, *Nematolebias whitei* and *Kryptolebias marmoratus*), which are results of a parallel work of our group (Gajardo et al., in prep.). Finally, we estimated the repetitive content of each genome using RepeatMasker [86] with the families of transposable elements (TEs) identified on each species.

### Comparative analysis of TEs

For the comparison of the contribution of the different superfamilies of TEs, we calculated the log_2_ Fold-change (log_2_FC) of the absolute number of base pairs associated with a given TE superfamily (TEsup) in a reference species (# bps_ref_), over the same value on every other species (# bps_sp_) (Eq. 1). We applied this methodology iteratively, such that every species was used as a reference once, and also every TE superfamily was compared.

$${\mathrm{log_2FC}}_{\mathrm{TEsup}}={\mathrm{log}}_2\left(\#\;{\mathrm{bps}}_{\mathrm{ref}}/\#\;{\mathrm{bps}}_{\mathrm{sp}}\right)$$

Additionally, we analyzed the number of TE fragments at different Kimura distances (K2P), a parameter calculated by RepeatMasker which is indicative of the time passed since a given insertional event occurred. By utilizing a custom workflow based on R and Snakemake (available at https://github.com/fgajardoe/TE-workflows), we visualized this information, in each species, in a TE order-specific manner, obtaining the Kimura distributions. In addition, we compared the TE content of superfamilies within the four species of *Orestias*, and also with the two closely related outgroups, *Anableps anableps* and *Cyprinodon variegatus*.

Finally, in order to gain insights into the evolutionary origins of the multiple superfamilies of TEs in this panel, we generated Venn diagrams to analyze the co-occurrence of superfamilies across the species using the InteractiVenn web service [87]. This allowed us to further explore the superfamilies of TEs found in *Orestias* regarding the other genera, and among the *Orestias* species themselves.

### Data collection for phylogenomic and phylogenetic analyses: taxa and genetic datasets

For the phylogenomic analyses, all the available Cyprinodontiformes genomes (26 species) were downloaded from the NCBI database (https://www.ncbi.nlm.nih.gov/genome), together with 3 outgroup species belonging to the two most closely related orders: *Odontesthes bonariensis* (Atheriniformes), *Xenentodon cancila* and *Oryzias woworae* (Beloniformes) [Supplementary 1, Table S4]. Additionally, we included another six genomes that were sequenced, assembled and annotated by our work group: the four previously mentioned species of the *Orestias* genus (*O. ascotanensis*, *O. chungarensis*, *O. gloriae* and *O. laucaensis*) and two species from the Aplocheiloidei suborder, *Austrolebias charrua* and *Cynopoecilus melanotaenia* (Gajardo et al. in prep.).

For the phylogenetic reconstruction of the order Cyprinodontiformes, we applied stratified taxa sampling [20], considering one species of every genus of every family of the order, and as many available nucleotide sequences. For this purpose, we performed two complementary database searches: one for the taxa dataset and other for the nucleotide sequences dataset. For the first one, we checked the taxonomic categories and members of the order in the databases of Eschmeyer’s Catalog of Fishes [88], Killi-Data (www.killi-data.org), and the Taxonomy database of NCBI (https://www.ncbi.nlm.nih.gov/taxonomy), and the validity of the specific name was then verified with the R package Rfishbase [89]. Next, we searched and downloaded the nucleotide sequences available in the NCBI database [90] for the previously gathered species. This is how we compiled a taxa dataset of 212 different species, comprising 13 of the 14 families of the order (the Pantanodontidae family was not included) and 14 outgroup species (6 Beloniformes, 3 Atheriniformes, 3 Cichliformes, 1 Blenniiformes, 1 Kurtiformes and 1 Gobiiformes species), representing the most complete taxa-set of the Cyprinodontiformes order for a phylogenetic analysis to date. The outgroup species were chosen on the availability of molecular markers for each taxa and also based on the previous phylogenetic relationships reported for the different fish orders [9, 47]. The sequences dataset contemplates a total of 12 markers, four mitochondrial genes (16S, *cox1*, *nd2* and *cytb*) and eight nuclear genes (*rag1*, *glyt*, *sreb2*, *rho*, *enc1*, *sh3px3*, *myh6* and *x-src*) for a total of 7,220 bp [Supplementary Table S7]. Furthermore, we downloaded the available mitochondrial genomes of the species included in the taxa-set, in order to complete the sequence dataset of the mitochondrial markers used in this study. These mitogenomes were also annotated using MitoAnnotator, from the MitoFish database [83].

Finally, in order to generate an updated map of the georeferenced distribution of the families belonging to the Cyprinodontiformes order, we searched for all the occurrences reported for this order in the GBIF database (https://www.gbif.org/), and generated a map using an R custom script (See the *“Availability of data and materials*” section).

### Phylogenomic reconstruction

For the phylogenomic reconstruction of the order Cyprinodontiformes, we performed a BUSCO analysis, using the -Cyprinodontiformes tag, for each of the 35 genomes in order to extract all the complete single-copy genes per species [Supplementary 1: Table S4]. Next, we selected only those orthologs that were found in all species, thus establishing the core set [Supplementary Table S6]. For each selected gene, we built a phylogenetic tree using RAxML v8.2.12 [91], using the GTRGAMMA model, and the node support was obtained using a bootstrap analysis of 1,000 pseudoreplicates. We rooted the tree using *Odonthestes bonariensis* as the outgroup. Finally, we coalesced all of them to obtain the species tree by using ASTRAL II v5.7.4 [92] with 1,000 bootstrap replicates. All the custom scripts used for this analysis are available on GitHub (See *“Availability of data and materials”* section in Methods).

### Phylogenetic reconstructions

We generated a multiple sequence alignment (MSA) with MAFFT v7.475 [93] for each of the 12 genes. Then, we extracted the conserved blocks with Gblocks v0.91b [94] in the semi-strict mode (controlled by the -h5 = h parameter). Later, we built a matrix of concatenated sequences using the 12 conserved blocks with the tool Catsequences [95], obtaining a total length of 7,220 bp.

Phylogenetic reconstructions were performed under the Bayesian Inference (BI) and Maximum Likelihood (ML) algorithms using MrBayes v3.2.7 [96] and RAxML v8.2.12 [91], respectively. For BI, we used PartitionFinder v2.1.1 [97] to estimate the substitution model for coding and non-coding partitions [Supplementary 1, Table S5]. For coding genes, we performed GeneWise v2.4 [98] alignments using reference amino acid sequences in order to recover the open reading frames on which the protein was coded, thus estimating substitution models for each codon position. The BI analysis was run three times with 200,000,000 generations, sampling every 20,000 iterations, and a consensus tree considering a burn-in of 25% was generated. The ML reconstruction was performed using the GTRGAMMA model, and the node support was obtained using a bootstrap analysis of 1,000 pseudoreplicates.

### Divergence time estimation

To estimate divergence times within the Cyprinodontiformes order, we performed a Bayesian inference analysis in BEAST v2.6.5 [99], using data from the fossil records and previous coalescent time estimations to calibrate the molecular clock [Supplementary 1, Table S3]. For the analysis, the best model of sequence evolution was previously selected using bModelTest [100]. We used a Birth–Death model and a relaxed Log-normal clock, running the analysis for 200,000,000 generations, sampling every 20,000 iterations, with a burn-in of 10%. The convergence of the results was analyzed in Tracer v1.7.1 [101] and the results were summarized in a single ultrametric tree using TreeAnnotator v2.5.1 [102].

### Supplementary Information

Supplementary Material 1.

Supplementary Material 2: Table S6: Set of 902 single-copy ortholog groups shared by the 35 taxa considered for the phylogenomic analysis.

Supplementary Material 3: Table S7: Complete dataset of molecular markers used for performing Bayesian Inference and Maximum likelihood phylogenetic reconstructions.

## Data Availability

The datasets supporting the conclusions of this article are available in the NCBI repository, under the accession number PRJNA907400 and in Zenodo under the 10.5281/zenodo.7400790. All the scripts, workflows, and parameters used to reproduce these results are available on GitHub (https://github.com/fgajardoe/Phylogenomic-analysis-of-killifishes). Mitogenomes nucleotide sequences and annotation were submitted to NCBI under the following accession numbers: PP097440 for *Orestias chungarensis*, PP097441 for *Orestias laucaensis* and PP101846 for *Orestias gloriae*. The data will be made available by the corresponding author upon a request.
