# Supplementary material for: Genomes of the Orestias pupfish from the Andean Altiplano shed light on their evolutionary history and phylogenetic relationships within Cyprinodontiformes
Source: BMC Genomics. 2024 Jun 18;25:614. doi: 10.1186/s12864-024-10416-w (PMC11184842; doi:10.1186/s12864-024-10416-w)
Supplement: Supplementary file 1 — Supplementary Material 1. [file 12864_2024_10416_MOESM1_ESM.pdf]

Genomes of the *Orestias* pupfish from the Andean Altiplano shed light on their evolutionary history and phylogenetic relationships within Cyprinodontiformes.

Pamela Morales, Felipe Gajardo, Camilo Valdivieso, Moisés A. Valladares, Alex Di Genova, Ariel Orellana, Rodrigo A. Gutiérrez, Mauricio González, Martín Montecino, Alejandro Maass, Marco A. Méndez, Miguel L. Allende.

SUPPLEMENTARY MATERIAL

ADDITIONAL FILE 1

INDEX

FIGURES

Figure S1......3

Figure S2......3

Figure S3......4

Figure S4......5

Figure S5......6

Figure S6......7

Figure S7......8

TABLES

Table S1......9

Table S2......10

Table S3......11

Table S4......12

Table S5......13

References......14

FIGURES

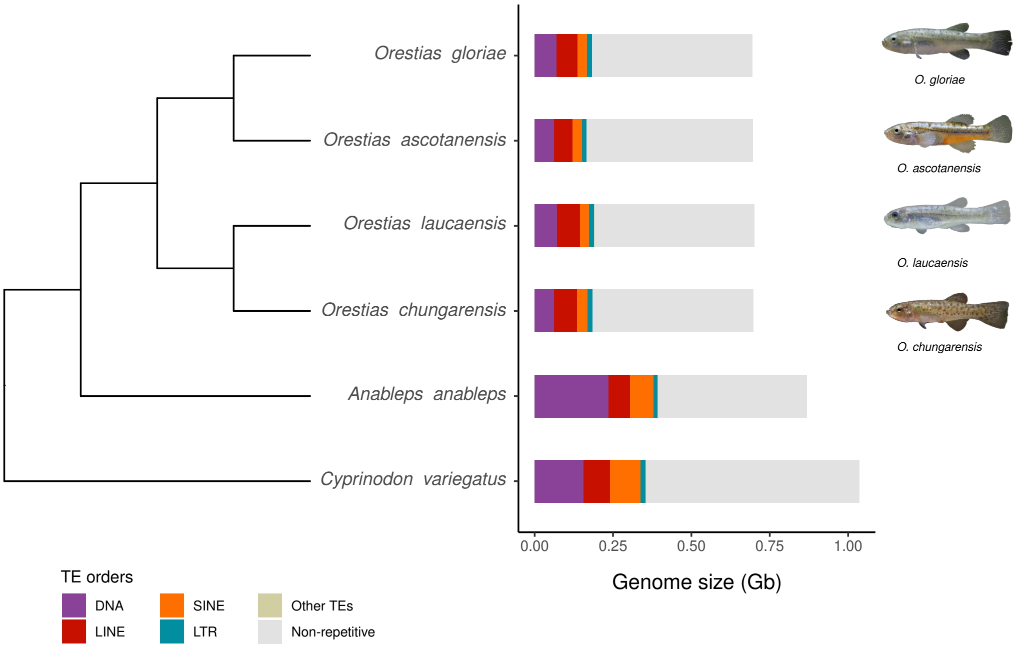

Figure S1.

The contribution of TEs to genome size. Left: Representation of the phylogenetic relationships (this study) of four *Orestias* species and their close relatives with available genomic information. Right: Barplot showing the contribution of the main orders of TEs (DNA elements, LINEs, SINEs, LTRs, other Tes) and non-repetitive DNA to the genome size.

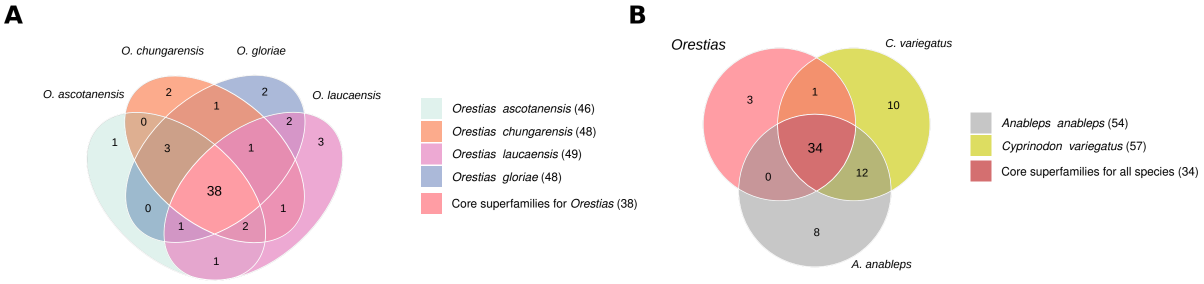

Figure S2.

(A) Venn diagram depicting the number of shared and exclusive TE superfamilies in genomes of the four *Orestias* species evaluated. The number in parenthesis corresponds to the total number of known superfamilies in each species.

(B) A Venn diagram indicating the number of shared and exclusive TE superfamilies considering the 38 superfamilies shared by the four *Orestias* species (indicated as "*Orestias*" in the diagram), and also those found in the genomes of their close relatives with available genomic information, *C. variegatus* and *A. anableps*. The number in parenthesis corresponds to the number of known superfamilies in each group.

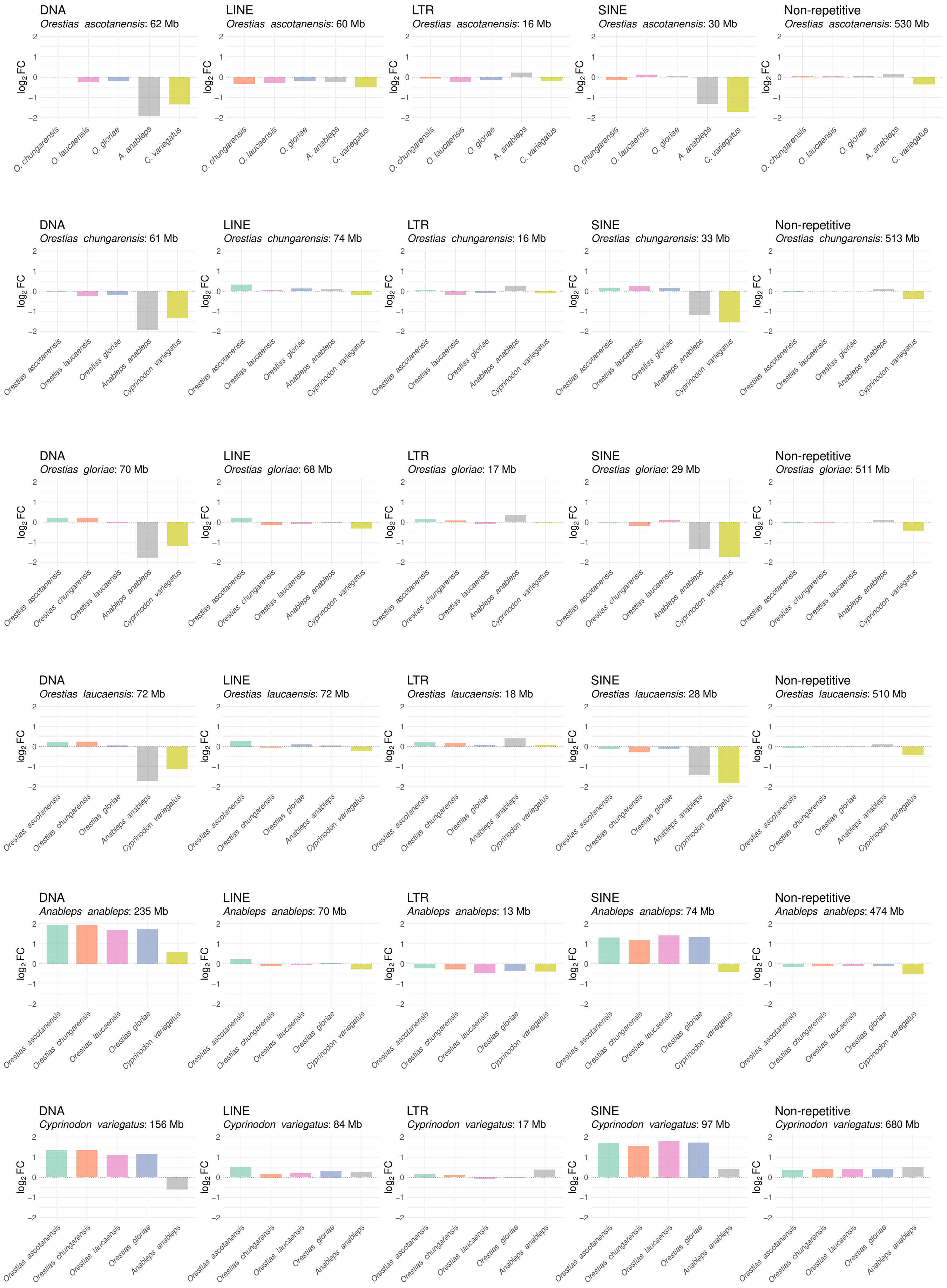

Figure S3.

Log_2 fold-change of the number of bases of the main orders of TE and non-repetitive regions in each species regarding every other species in the panel.

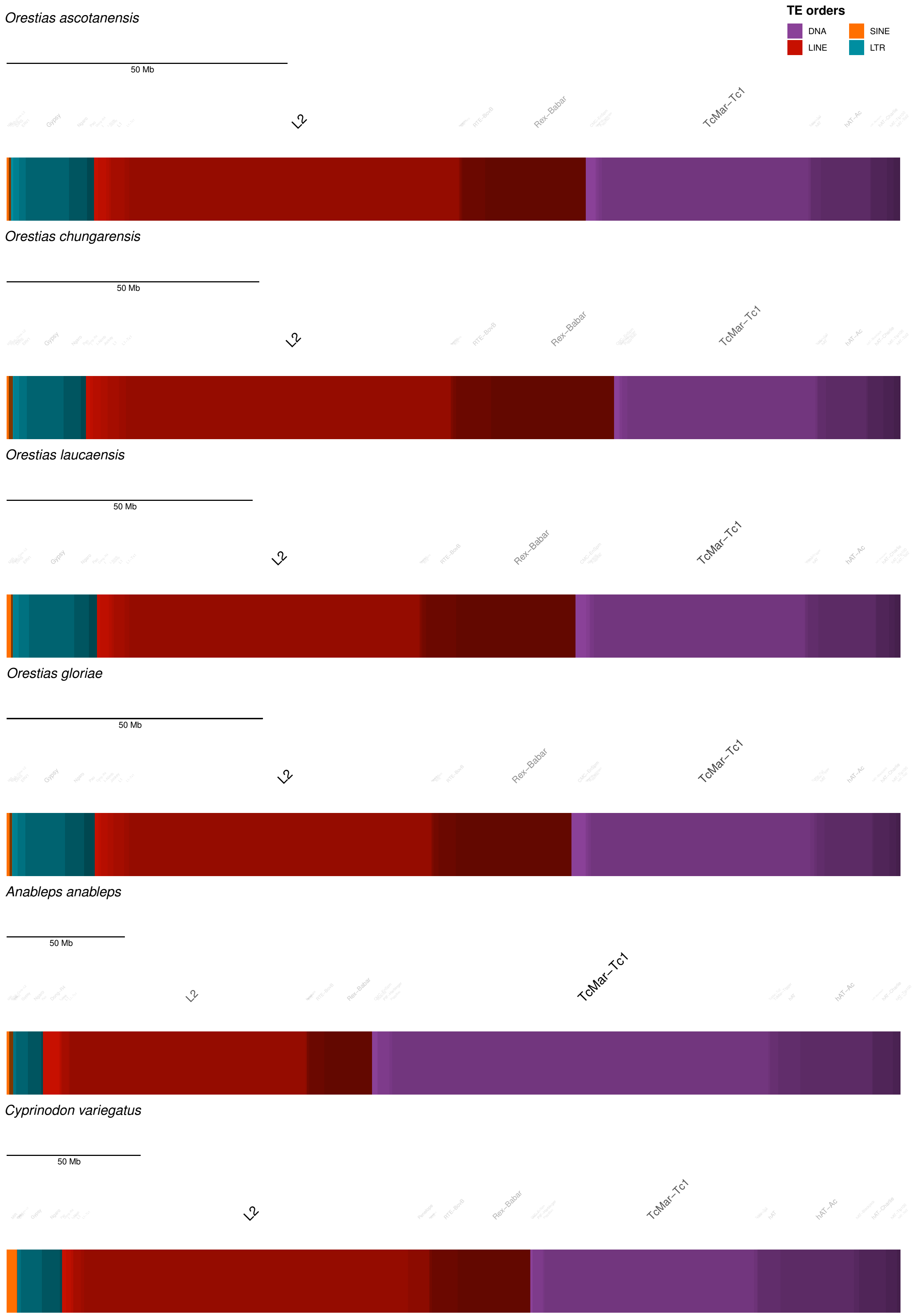

Figure S4.

Proportional contribution of the core set of TE superfamilies to the repetitive fraction of the genomes.

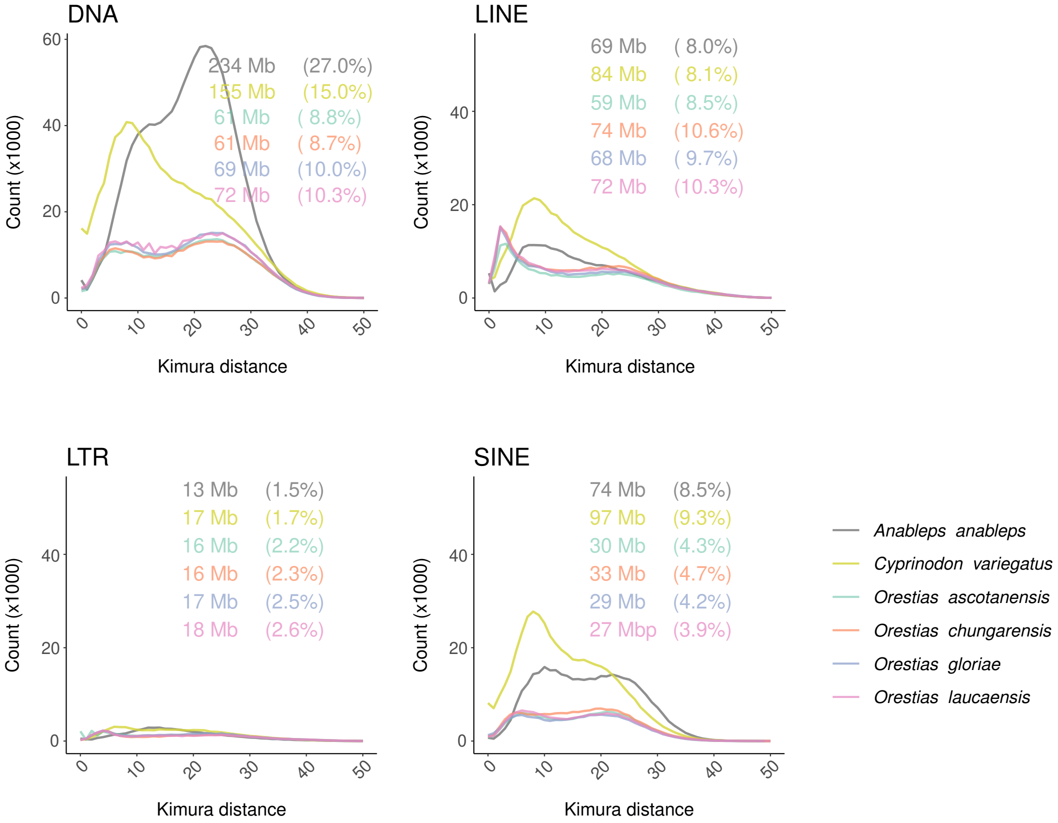

Figure S5.

Kimura distance distributions of the most abundant orders of TE (LINEs, SINEs, LTRs and DNA elements) for the four *Orestias* species and their close relatives with available genomic information, *C. variegatus* and *A. anableps*. Total accumulated base pairs and the percentage of contribution (in parenthesis) to the genome size of each species are shown for each category.

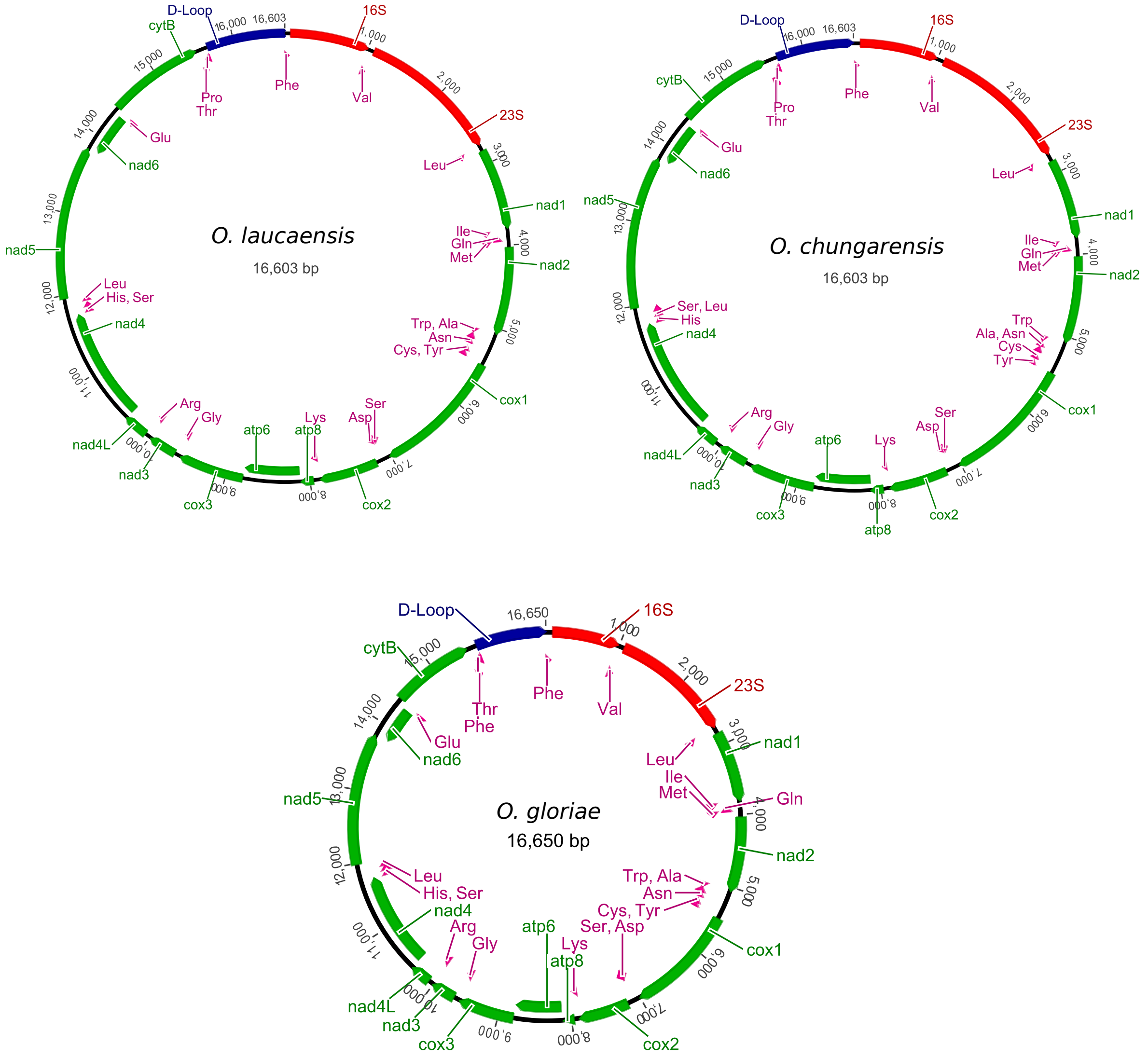

Figure S6.

Representation of the mitogenome of *O. laucaensis*, *O. chungarensis*, and *O. gloriae*.

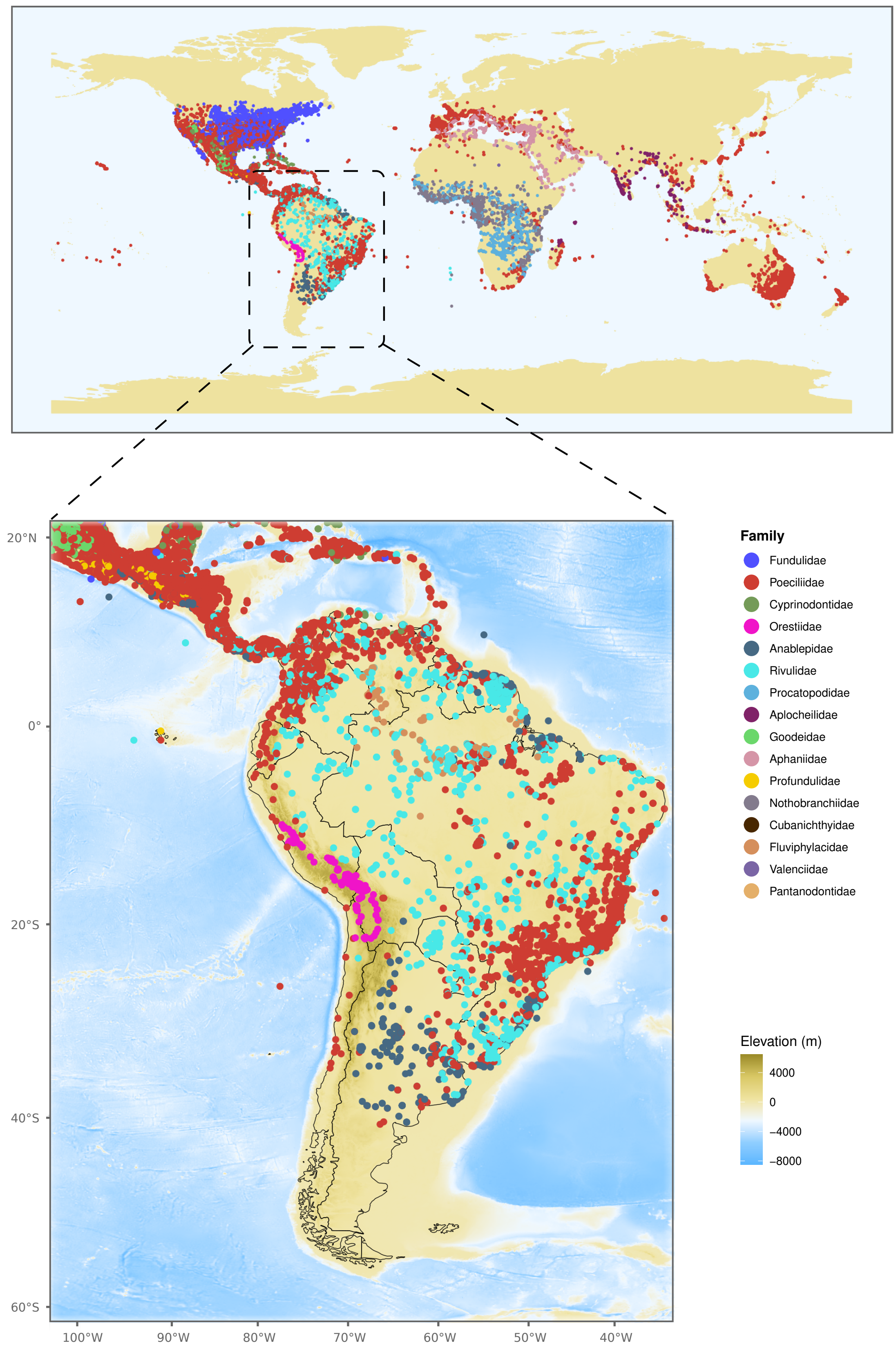

Figure S7.

Geographic distribution map for the families of Cyprinodontiformes order in the world. The families indicated are those suggested in this study (see the text for details). The dashed box is a zoomed area of South America highlighting the distribution of the Orestiidae family.

TABLES

Table S1.

Metrics for genome assemblies and gene annotation.

	O. ascotanensis	*O. gloriae*	*O. laucaensis*	*O. chungarensis*
Assembly length (only contigs)	670,593,887	690,600,134	694,856,734	693,821,163
Assembly length (scaffolds)	696,398,878	694,856,734	700,593,382	698,304,763
Total number of contigs	25,067	37,838	44,473	41,074
Total number of scaffolds	2,394	6,964	8,573	7,858
Contigs in scaffolds	23,746	37,170	43,564	40,472
Contigs in scaffolds (%)	94.7	98.2	98.0	98.5
Longest scaffold	14,017,584	13,816,820	13,868,713	13,882,505
N50 contig	60,975	29,061	26,811	28,026
N50 scaffold	2,674,408	2,661,878	2,605,576	2,617,268
Total number of predicted genes	33,429	31,270	31,026	31,168
Number of genes in ortholog groups	32,980 (99.0%)	30,811 (98.5%)	30,601 (98.6%)	30,685 (98.4%)
Source	*Di Genova et al., 2022*	*This study*	*This study*	*This study*

Table S2.

Comparison of assembly metrics before and after the reference-guided scaffolding.

	Reference	Before reference-guided scaffolding			After reference-guided scaffolding		
	O. ascotanensis	***O. gloriae***	***O. laucaensis***	***O. chungarensis***	***O. gloriae***	***O. laucaensis***	***O. chungarensis***
Assembly length (contigs)	670,593,887	690,600,134	695,845,882	693,821,163	690,600,134	694,856,734	693,821,163
Assembly length (scaffolds)	696,398,878	691,769,334	697,003,382	694,983,163	694,856,734	700,593,382	698,304,763
N50 contig	60,975	29,061	26,811	28,026	29,061	26,811	28,026
N50 scaffold	2,674,408	61,559	53,112	57,253	2,661,878	2,605,576	2,617,268
Longest scaffold	14,017,584	462,068	590,328	508,560	13,816,820	13,868,713	13,882,505
Total number of contigs	25,067	37,838	44,473	41,074	37,838	44,473	41,074
Number of reordered contigs	-	-	-	-	37,170 (98.2%)	43,564 (98.0%)	40,472 (98.5%)
Total number of scaffolds	2,394	37,838	44,473	41,074	6,964	8,573	7,858
BUSCO (%)							
Complete	90	78.4	77.3	78.2	89.9	89.7	89.7
Single	89.7	78.1	76.9	77.9	89.5	89.2	89.3
Duplicated	0.3	0.3	0.4	0.3	0.4	0.5	0.4

Table S3.

Fossil records used to calibrate the time-calibrated phylogenetic inference obtained using BEAST.

ID	Name	Type	Distribution	Mean	SD	Offset	Reference
A	Diversification (Beloniformes,(Atheriniformes, Cyprinodontiformes)	Secondary calibration	Normal	77.40	5.5	0	Betancur et al, 2017
B	Diversification (Atheriniformes,Cyprinodontiformes)	Secondary calibration	Normal	76.691	5.5	0	Betancur et al, 2017
C	Cyprinodontiformes	Secondary calibration	Normal	70.720	5.5	0	Capobianco & Friedman, 2019
D	Cyprinodontoidei	Secondary calibration	Normal	46.270	5.2	0	Capobianco & Friedman, 2019
E	Cyprinodon	Fossil	Log Normal	3.955	1	2.58	Miller, 1945; Helmstetter, 2017

Table S4.

Accession number and identifier for the genome assemblies used in the phylogenomic analysis.

**Species**	**Assembly ID**	**Accession number**
Anableps anableps	fAnaAna1.pri	GCA_014839685.1
Aphyosemion australe	MPIBA_Aaus_1.0	GCA_006937985.1
Austrofundulus limnaeus	Austrofundulus_limnaeus-1.0	GCF_001266775.1
Callopanchax toddi	MPIBA_Ctod_1.0	GCA_006937965.1
Cyprinodon nevadensis pectoralis	ASM77601v1	GCA_000776015.1
Cyprinodon tularosa	ASM1607723v1	GCF_016077235.1
Cyprinodon variegatus	C_variegatus-1.0	GCF_000732505.1
Fundulus heteroclitus	MU-UCD_Fhet_4.1	GCF_011125445.2
Gambusia affinis	ASM309773v1	GCA_003097735.1
Gambusia holbrooki	ASM966307v1	GCA_009663075.1
Kryptolebias hermaphroditus	ASM789654v1	GCA_007896545.1
Kryptolebias marmoratus	ASM164957v2	GCF_001649575.2
Nematolebias whitei	NemWhi1	GCF_014905685.1
Nothobranchius furzeri	Nfu_20140520	GCF_001465895.1
Nothobranchius kuhntae	MPIBA_Nkuh_1.0	GCA_006942095.1
Pachypanchax playfairii	MPIBA_Ppla_1.0	GCA_006937955.1
Poecilia formosa	Poecilia_formosa-5.1.2	GCF_000485575.1
Poecilia latipinna	P_latipinna-1.0	GCF_001443285.1
Poecilia mexicana	P_mexicana-1.0	GCF_001443325.1
Poecilia reticulata	Guppy_female_1.0+MT	GCF_000633615.1
Poeciliopsis occidentalis	ASM1088303v1	GCA_010883035.1
Poeciliopsis retropinna	ASM1027707v1	GCA_010277075.1
Poeciliopsis turrubarensis	ASM1027701v1	GCA_010277015.1
Xiphophorus couchianus	X_couchianus-1.0	GCF_001444195.1
Xiphophorus hellerii	Xiphophorus_hellerii-4.1	GCF_003331165.1
Xiphophorus maculatus	X_maculatus-5.0-male	GCF_002775205.1
Orestias ascotanensis	ASM1293152v1	GCA_012931525.1
Orestias gloriae	ASM3287625v1	GCA_032876255.1
Orestias laucaensis	ASM3287626v1	GCA_032876265.1
Orestias chungarensis	ASM3287630v1	GCA_032876305.1
Austrolebias charrua	--	--
Cynopoecilus melanotaenia	--	--
Odontesthes bonariensis	GWU_Obon_1.1	GCA_014825785.1
Xenentodon cancila	fXenCan1.pri	GCA_014839995.1
Oryzias woworae	OryWow_0.9	GCA_016861445.1

Table S5.

Models predicted by PartitionFinder for partitions corresponding to the 12 molecular markers used in the Bayesian inference with MrBayes.

Subset	Best Model	# sites	subset id	Partition names
1	GTR+I+G	381	67c0b9a2f00d0a8274f53be52aaae148	16S_pos1
2	TVM+I+G	184	cc54b7659c4225472f63763dbc9fe378	COX1_pos2
3	TRN+G	183	14d84fc9c8e562e2f404fa88da86990d	COX1_pos3
4	GTR+I+G	183	841bb2b4a5bc78333de65f1940741edc	COX1_pos1
5	GTR+I+G	274	beb472eb79dec4fa2078e88159a9216a	RHO_pos2
6	TRNEF+G	274	aeea7864c11c4f094c52d5677831c16a	RHO_pos3
7	TIMEF+I+G	274	b962a4ec3be744765035041ed5689f05	RHO_pos1
8	GTR+I+G	429	06c6a319e44feed25906b4fc78c2d164	ENC1_pos1, SREB2_pos1
9	TVM+I+G	229	29698d1d5a21d92a17a85576a9c65e14	ENC1_pos2
10	SYM+I+G	228	e62bd32e2ce0cafc75cddc7247a4488f	ENC1_pos3
11	GTR+I+G	114	06b6bca9d68a8a3d6fa7cb2293d64492	CYTB_pos2
12	GTR+I+G	114	e8ed8cdcd9da80912921c11a76c306ed	CYTB_pos3
13	SYM+I+G	113	7d9ac00753efcd038e4afefc407677db	CYTB_pos1
14	F81+I	200	74ac9f34bf86b45ecf1e5d955fa598c0	SREB2_pos2
15	GTR+I+G	199	fdad75c955fd92e7ec17044664744975	SREB2_pos3
16	GTR+I+G	198	b66092b2ef9182cd81dec7438134e56a	GLYT_pos2
17	K80+I+G	198	c111f06aa483591c358d4aae9fd10ef5	GLYT_pos3
18	TVM+G	198	71d7586f9dad2a87bdb645f2c82428d0	GLYT_pos1
19	GTR+I+G	594	a05330c9306283c5f50046913e0ab679	SH3PX3_pos1, MYH6_pos1, RAG1_pos1
20	GTR+I+G	377	c61cd3578fe9531975eace143efd011a	RAG1_pos2, MYH6_pos2
21	TVMEF+G	234	58aa4fb3740416de62d73ea7b58aa37b	MYH6_pos3
22	GTR+G	339	e5e40a8b607a217d2ee1281d664b6ffe	ND2_pos3
23	TVM+I+G	339	e72269c8dce5198f7f91ff539d8fdb45	ND2_pos1
24	TVM+I+G	339	aca1e55e61c7063c4998129b85f03721	ND2_pos2
25	TVMEF+I+G	142	4ad41841a47b84423fbeceeb4d309340	RAG1_pos3
26	GTR+I+G	215	3461ec8895d7289e96379a5af815fafe	SH3PX3_pos2
27	GTR+I+G	215	a910609dd0ed0feb09046a4e2474f2cf	SH3PX3_pos3
28	TVM+I+G	151	ffa9dedf612d405dff74e6a2c60e1706	X-SRC_pos3
29	SYM+I+G	151	a12c0a2c1e6120ba82a1d8538432a739	X-SRC_pos1
30	GTR+I+G	151	2f132856784dc1235d26b41f3973877b	X-SRC_pos2

References.

Di Genova A, Nardocci G, Maldonado-Agurto R, Hodar C, Valdivieso C, Morales P, et al. Genome sequencing and transcriptomic analysis of the Andean killifish *Orestias ascotanensis* reveals adaptation to high-altitude aquatic life. Genomics. 2022;114(1):305–315. doi:10.1016/j.ygeno.2021.12.018.

Miller, RR. Four new species of fossil cyprinodont fishes from eastern California. J. Wash. Acad. Sci. 1945;35(10):315–321.

Helmstetter AJ, Papadopulos AST, Igea J, Van Dooren TJM, Leroi AM, Savolainen V. Viviparity stimulates diversification in an order of fish. Nat Commun. 2016;7:11271. doi:10.1038/ncomms11271.

Capobianco A, Friedman M. Vicariance and dispersal in southern hemisphere freshwater fish clades: a palaeontological perspective. Biol Rev. 2019;94(2):662–699. doi:10.1111/brv.12473.

Betancur RR, Wiley EO, Arratia G, Acero A, Bailly N, Miya M, et al. Phylogenetic classification of bony fishes. BMC Evol Biol. 2017;17:162. doi:10.1186/s12862-017-0958-3.
